# Supplementary material for: Differentiation dynamics of mammary epithelial cells revealed by single-cell RNA sequencing
Source: Nat Commun. 2017 Dec 11;8:2128. doi: 10.1038/s41467-017-02001-5 (PMC5723634; doi:10.1038/s41467-017-02001-5)
Supplement: Supplementary file 1 — Supplementary Information [file 41467_2017_2001_MOESM1_ESM.pdf]

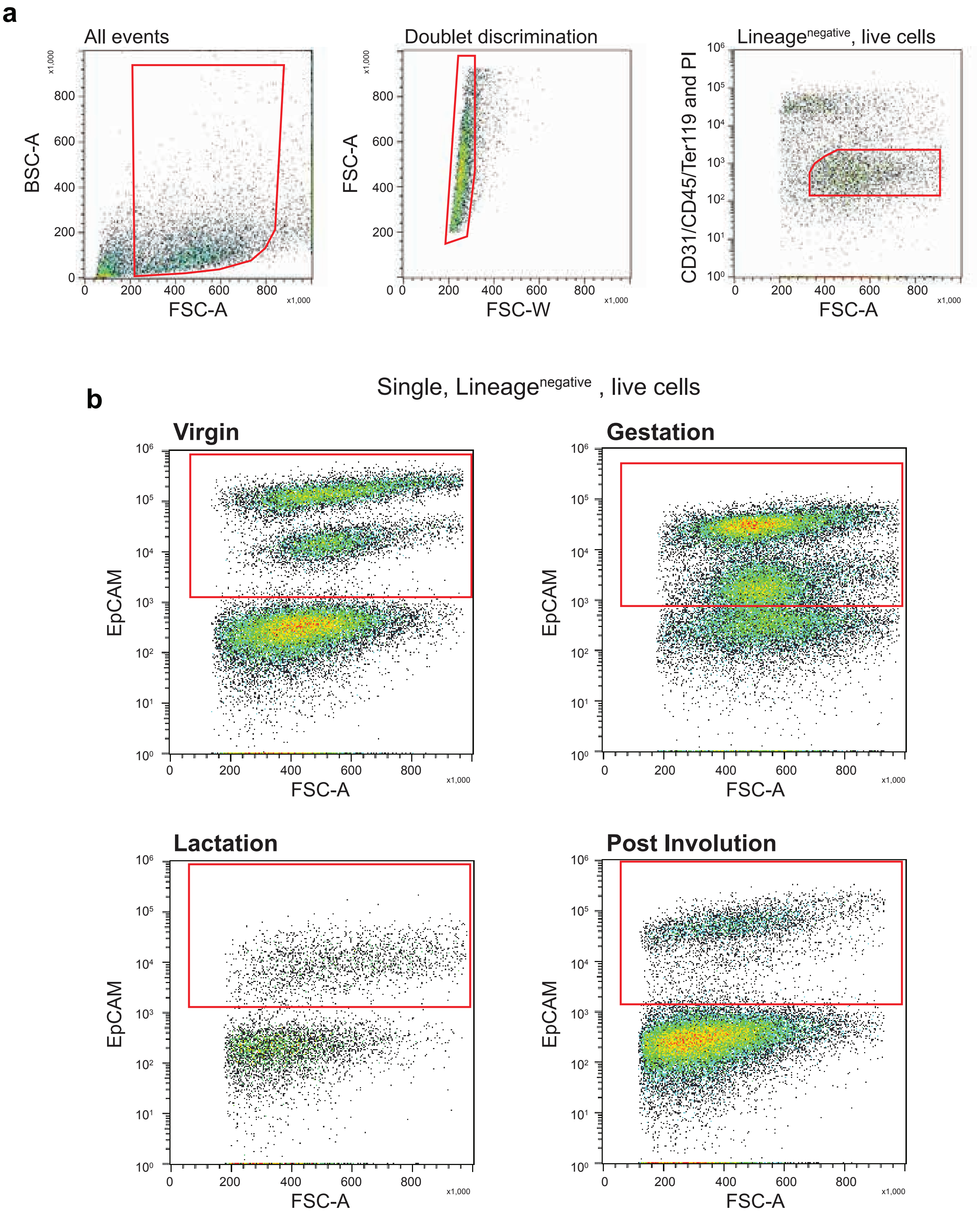

Supplementary Figure 1. Gating strategy to isolate MECs
(a) Gating strategy used to select live, lineagenegative singlets. (b) Representative plots for each of the four time-points are shown. FSC-W: forward scatter width, FSC-A: forward scatter area, BSC-A: backward scatter area.

a

SampleID	Number of cells	Total molecules	Genes Detected	NumberOfReads	Saturation
NP1	2249	9416.0	2843.0	202622976	72.7
NP2	2127	9501.0	2705.0	207363112	76.8
G1	2915	9622.0	2716.0	150459338	48.3
G2	3106	7816.5	2491.5	140968140	47.0
L1	5906	3767.5	1489.0	216771984	76.1
L2	3697	4260.0	1622.0	183422822	78.8
PI1	1500	7146.0	2366.0	78919991	64.2
PI2	4306	5845.0	2093.0	118933879	49.1

b

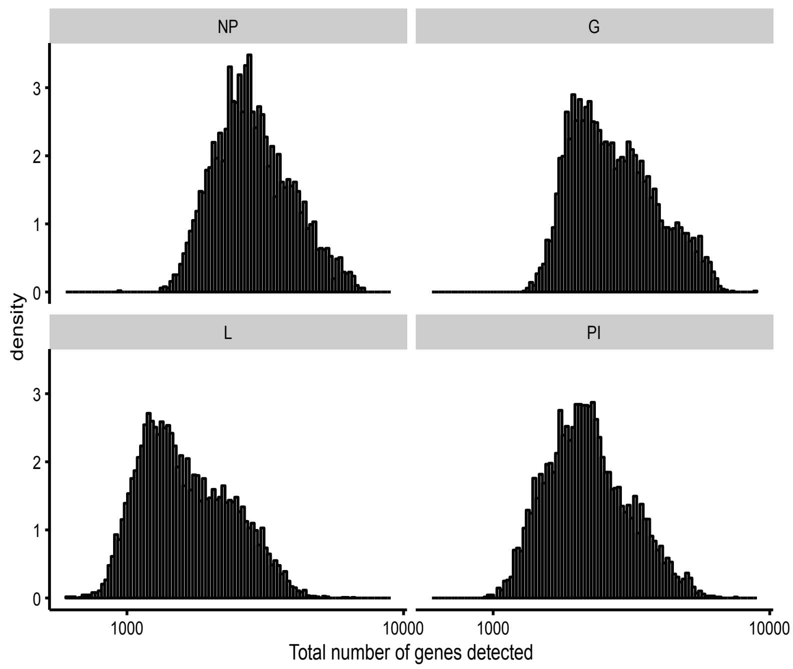

c

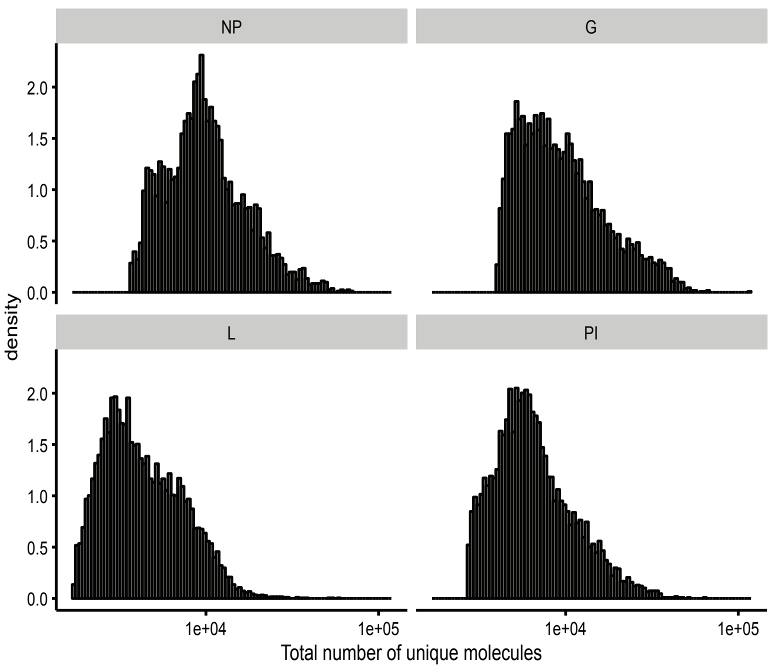

d

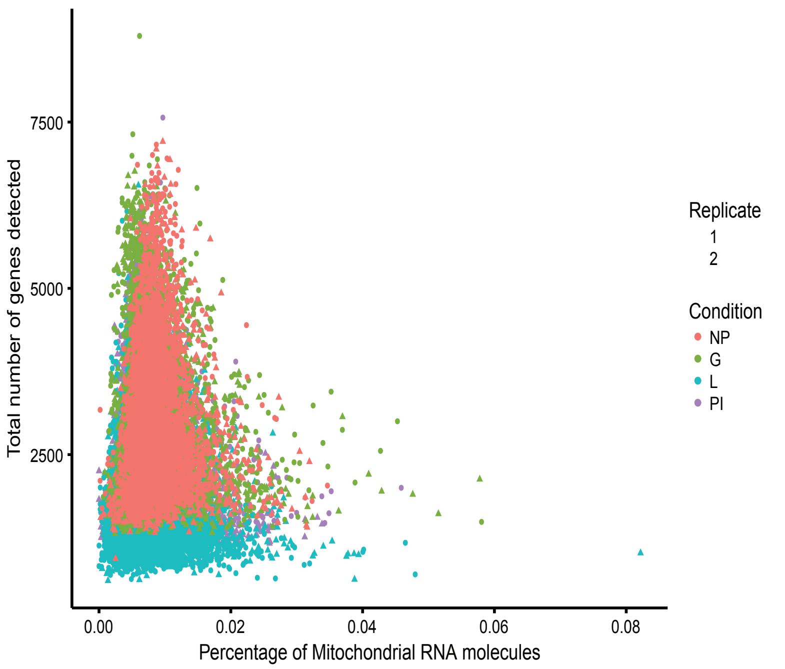

Supplementary Figure 2. Quality control of sequencing data
(a) Table summarising quality control criteria per sample. Number of unique molecules, genes detected and number of reads represent the median value for each sample. (b-c) Histograms for the four conditions showing the distributions of the number of genes detected (b) or total number of molecules (c). (d) Scatterplot of number of genes detected versus percentage of mitochondrial RNA molecules. For choice of thresholds see materials and methods.

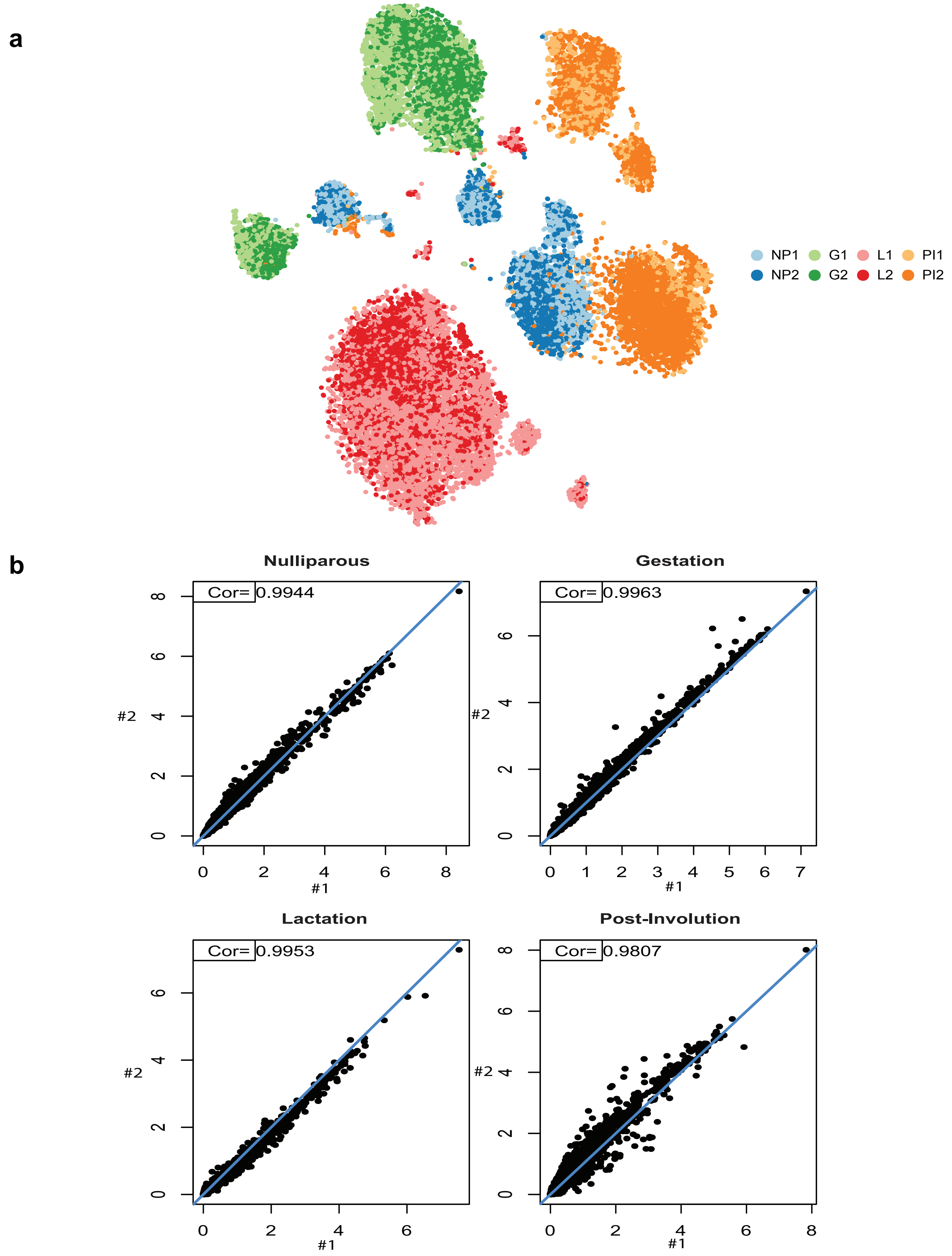

Supplementary Figure 3. Biological replicates account for little structure in the data
(a) t-SNE plot coloured by the eight different samples. All biological replicates are well mixed and none of the identified clusters is composed of cells from only one replicate. (b) Correlation of log transformed mean expression values of biological replicates for all four time-points. The identity line is represented in blue.

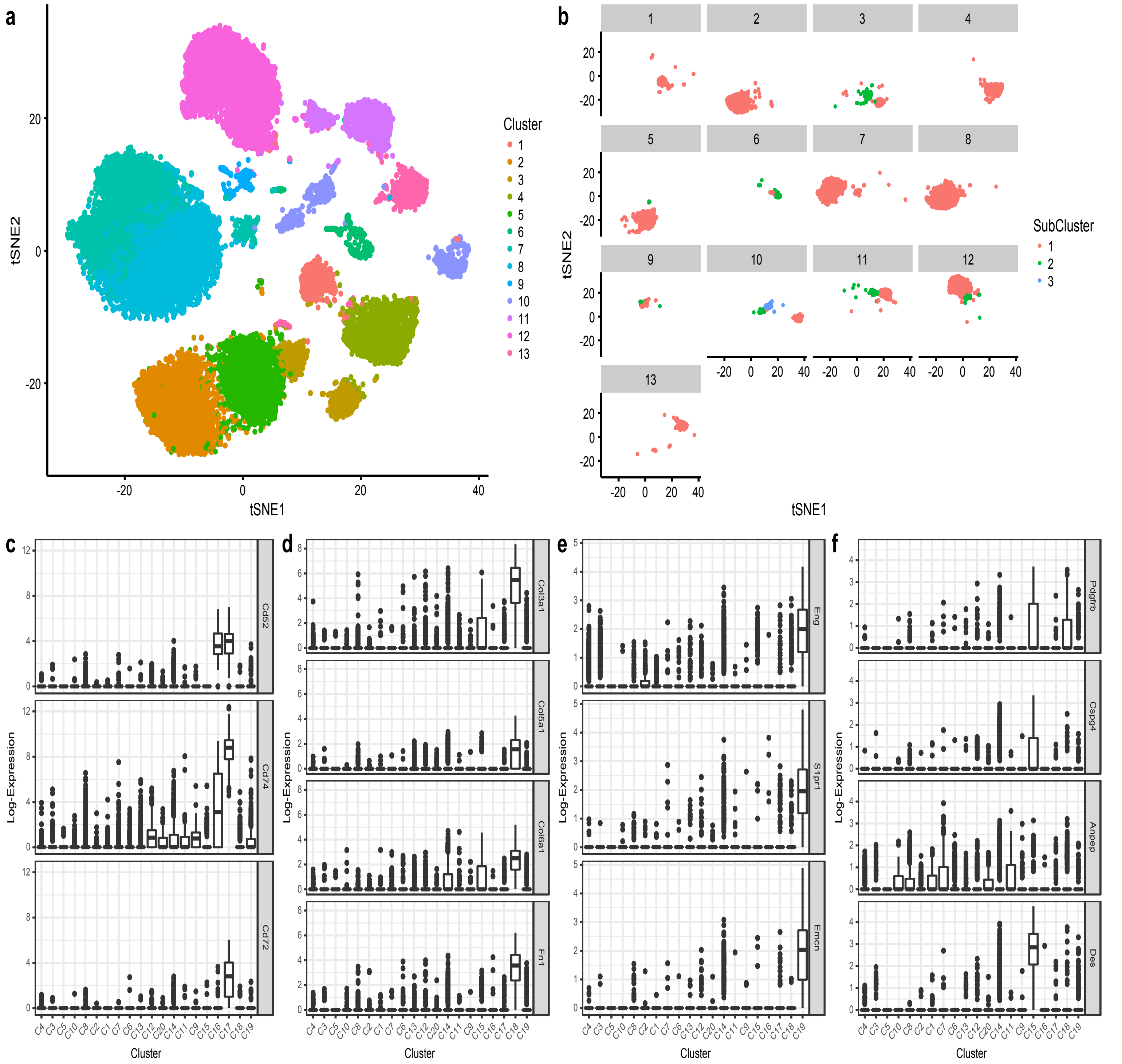

Supplementary Figure 4. Clustering strategy

(a) First round of clustering using an SNN-Graph and modularity maximization for cluster identification. Cluster numbers are unrelated to the naming scheme used in the manuscript. (b) Second round of clustering using hierarchical clustering based on Spearman's rank correlation with average linkage. The gap statistic was used as optimization criterion to choose k. (c-f) Marker genes that were used to remove immune cells (c), fibroblasts (d) and endothelial (e) cells from the analysis. Cluster C15 (Prc) showed some degree of expression for some pericyte markers (f). The centre line of the boxplots represents the median, the upper and lower hinges the first and third quartile and the whiskers extend to the largest or smallest value no further than 1.5 times the inter-quartile range from the hinge. Data points beyond this range are highlighted as dots.

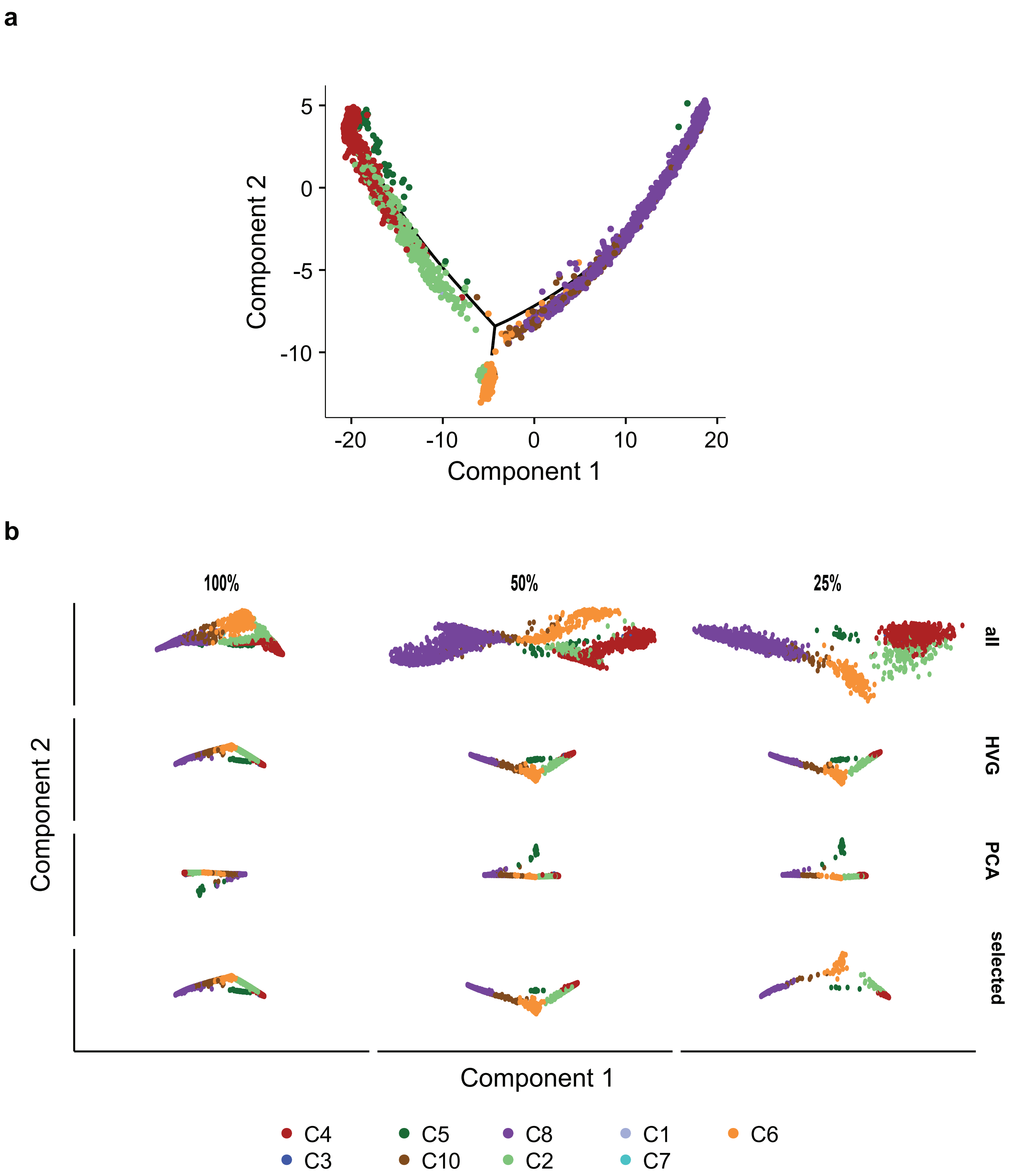

Supplementary Figure 5. Robustness of bifurcation event in diffusion map
(a) The differentiation trajectory as determined by Monocle (see materials and methods) coloured by cluster assignment. (b) The diffusion map is robust to down-sampling of cells (100, 50 or 25% of all cells were used in the left, middle or right panel, respectively) as well as the method of feature selection (all= all genes with mean expression level above 0.1, HVG= highly variable genes, PCA= first 50 components of PCA, selected= a manually selected choice of genes that are known to be involved in luminal cell differentiation). The gene list included: Csn2, Gata3, Prlr, Elf5, Esr1, Pgr, Aldh1a3, Wap, Tspan8, Krt18, Krt8, Areg, Fgfr1, Fgfr2, Notch1, Notch3, Foxc1 and Zeb2.

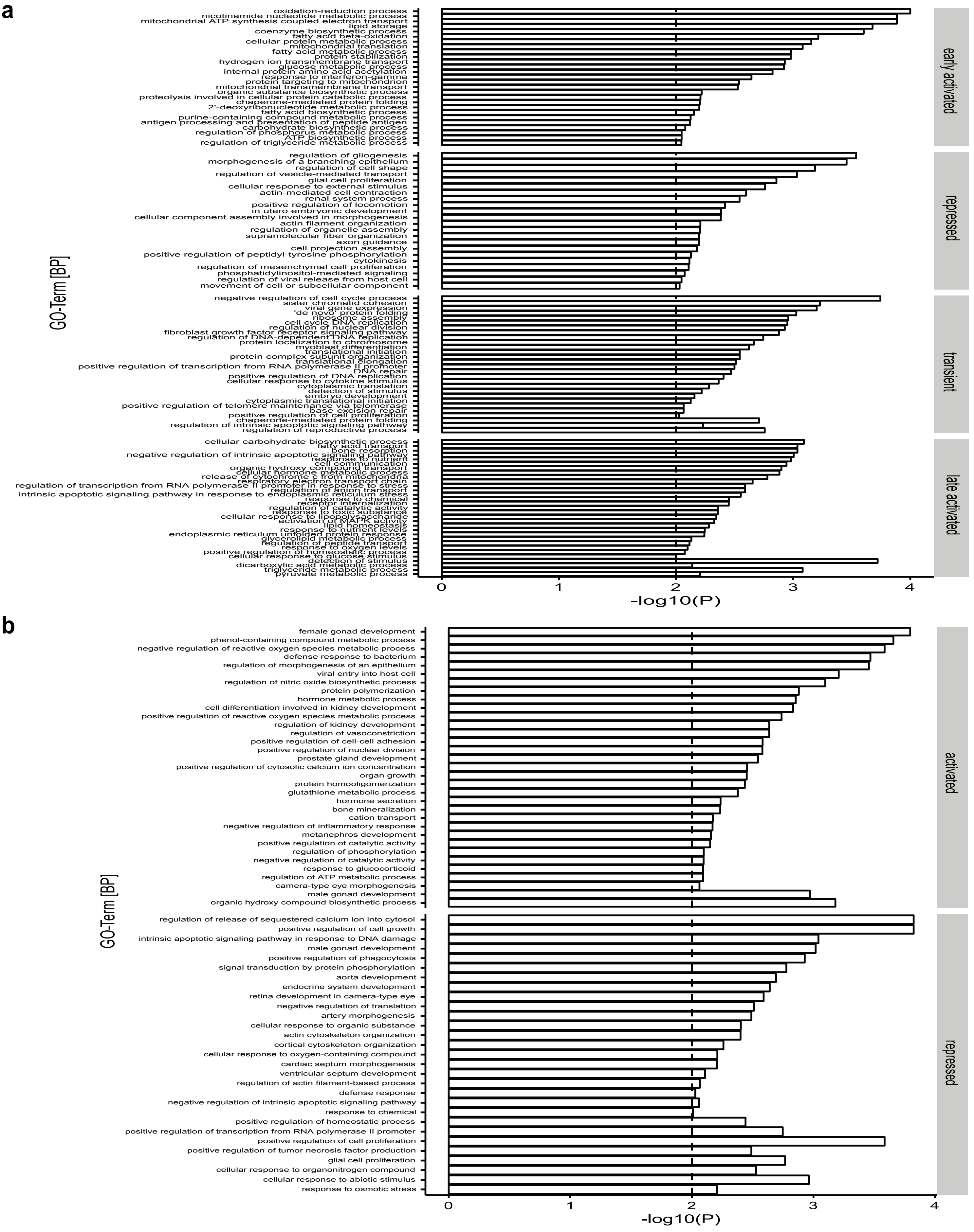

Supplementary Figure 6. Gene set enrichment analysis of pseudo-time dependent genes
(a) Significantly enriched GO-Terms for genes in the four clusters of the secretory branch. (b) Significantly enriched GO-Terms for genes in the two clusters of the hormone-sensing branch.

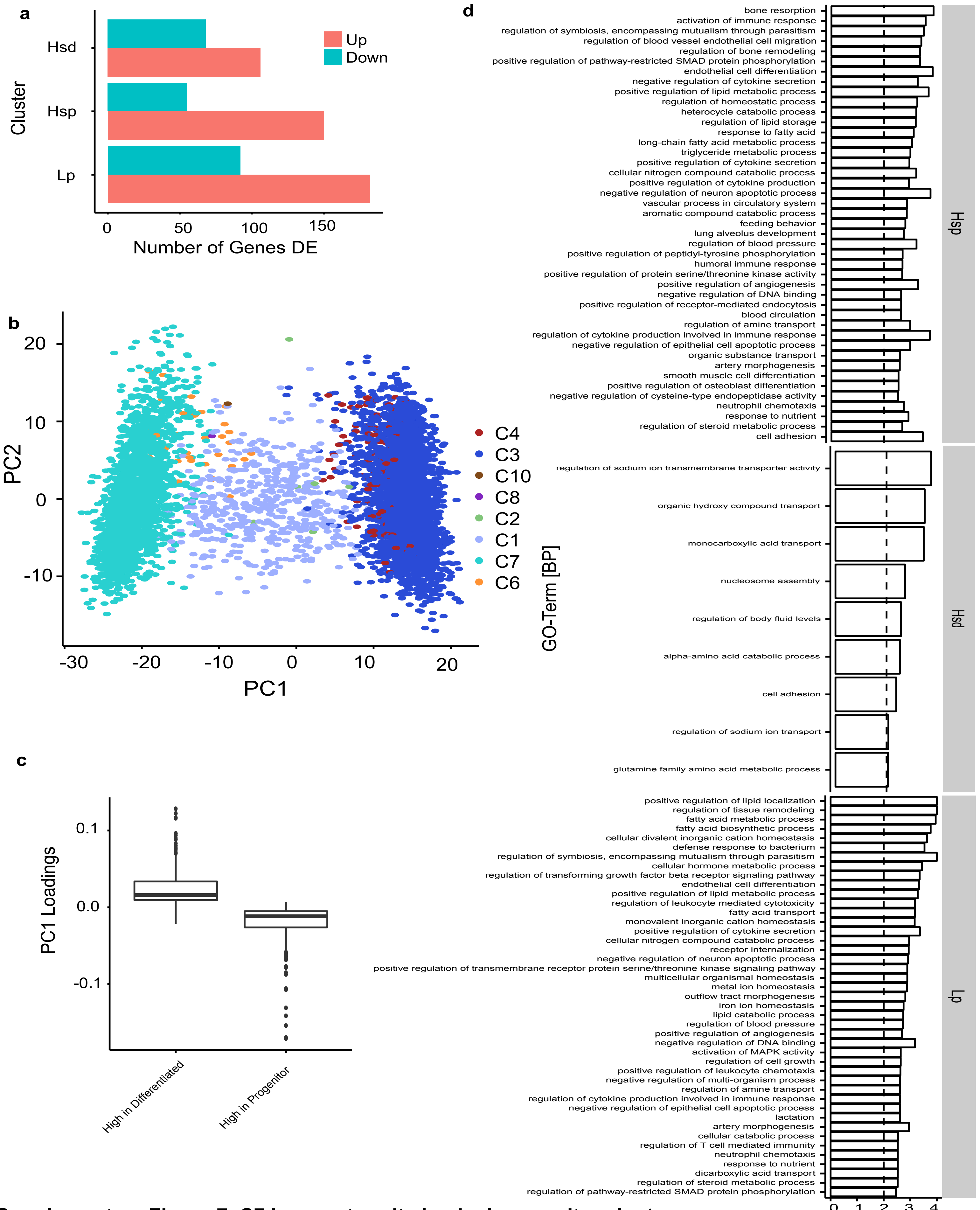

Supplementary Figure 7. C7 is a post-parity luminal progenitor cluster

(a) Number of differentially expressed genes between the NP and PI clusters of Hsd (C1 vs C2), Hsp (C3 vs C4) and Lp (C7 vs. C6). (b) Principal component analysis (PCA) on all luminal cells from the PI time-point computed on the top 500 differentially expressed genes between Lp and the rest of the luminal NP gland. (c) Loadings of the first components for genes that are high in differentiated or progentior cells. (d) Top GO-terms (biological processes) that are significantly enriched in upregulated genes from the differential expression analysis visualized in (a). Dashed line indicates P value threshold at 0.001.The centre line of the boxplot in (c) represents the median, the upper and lower hinges the first and third quartile and the whiskers extend to the largest or smallest value no further than 1.5 times the inter-quartile range from the hinge.

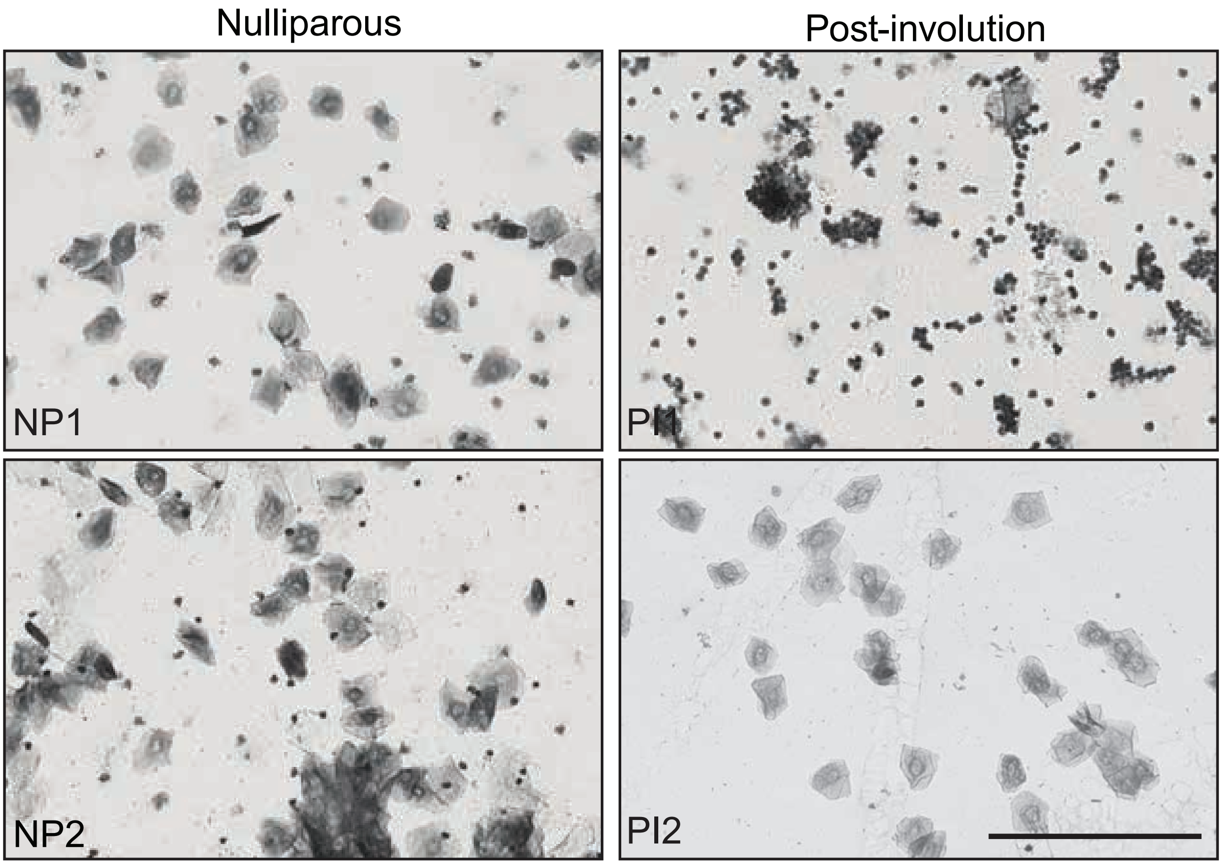

Supplementary Figure 8. Estrus cycle Vaginal smears of the mice from the NP and PI time-points
The animals were classified as being in estrus (NP1, NP2, PI2) or diestrus (PI1).
The scale bar represents 200µm.

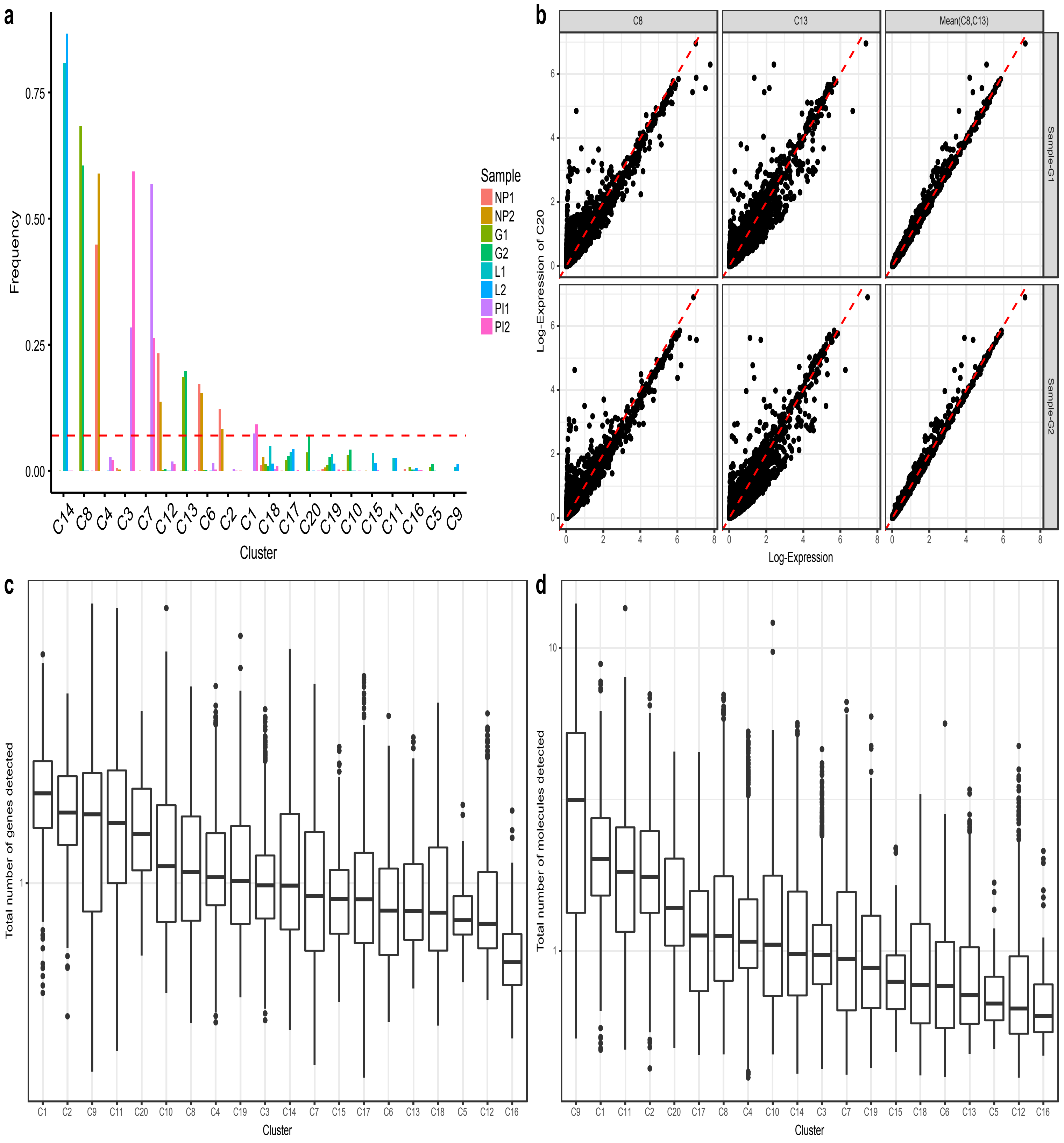

Supplementary Figure 9. Removal of doublet clusters

(a) Frequency of each cluster in all 8 samples, the threshold of 7% is indicated by the red dashed line. (b) Correlation of C20 (on the y-axes) with C8 (left panels), C13 (center panel) and the mean of C8 and C13 (right panel) for both samples G1 and G2. (c-d) Number of genes detected (c) and total number of molecules (d). The values for each cell were normalised to the median value of the sample in which the cell was captured. The boxplots were constructed as follows, the centre line represents the median, the upper and lower hinges the first and third quartile and the whiskers extend to the largest or smallest value no further than 1.5 times the inter-quartile range from the hinge. Data points beyond this are highlighted as dots.
