# Supplementary material for: Differentiation dynamics of mammary epithelial cells revealed by single-cell RNA sequencing
Source: Nat Commun. 2017 Dec 11;8:2128. doi: 10.1038/s41467-017-02001-5 (PMC5723634; doi:10.1038/s41467-017-02001-5)
Supplement: Supplementary file 3 — Description of Additional Supplementary Files [file 41467_2017_2001_MOESM3_ESM.docx]

**Description of Additional Supplementary Files**

File Name: Supplementary Data 1

Description: Differentially expressed genes between clusters.

File Name: Supplementary Data 2

Description: Differentially expressed genes between clusters.

File Name: Supplementary Data 3

Description: Differentially expressed genes between clusters.

File Name: Supplementary Data 4

Description: Differentially expressed genes between clusters.

File Name: Supplementary Data 5

Description: Differentially expressed genes between clusters.

File Name: Supplementary Data 6

Description: Differentially expressed genes between clusters.

File Name: Supplementary Data 7

Description: Differentially expressed genes between clusters.

File Name: Supplementary Data 8

Description: Differentially expressed genes between clusters.

File Name: Supplementary Data 9

Description: Differentially expressed genes between clusters.

File Name: Supplementary Data 10

Description: Differentially expressed genes between clusters.

File Name: Supplementary Data 11

Description: Differentially expressed genes between clusters.

File Name: Supplementary Data 12

Description: Differentially expressed genes between clusters.

File Name: Supplementary Data 13

Description: Differentially expressed genes between clusters.

File Name: Supplementary Data 14

Description: Differentially expressed genes between clusters.

File Name: Supplementary Data 15

Description: Differentially expressed genes between clusters.

File Name: Supplementary Data 16

Description: List of genes with pseudo-time dependent gene expression with same overall trend in the two branches.

File Name: Supplementary Data 17

Description: List of genes with pseudo-time dependent gene expression with different trends in the two branches1
